# Electrochemical Analysis for Demonstrating CO Tolerance of Catalysts in Polymer Electrolyte Membrane Fuel Cells

**DOI:** 10.3390/nano9101425

**Published:** 2019-10-08

**Authors:** Jiho Min, A. Anto Jeffery, Youngjin Kim, Namgee Jung

**Affiliations:** Graduate School of Energy Science and Technology (GEST), Chungnam National University, 99 Daehak-ro, Yuseong-gu, Daejeon, 34134, Korea; mjh9780@naver.com (J.M.); jeffeeanto@gmail.com (A.A.J.); yoongjin123@naver.com (Y.K.)

**Keywords:** chronoamperometry, CO tolerance, hydrogen oxidation reaction, PtRu alloy, evaluation protocol

## Abstract

Since trace amounts of CO in H_2_ gas produced by steam reforming of methane causes severe poisoning of Pt-based catalysts in polymer electrolyte membrane fuel cells (PEMFCs), research has been mainly devoted to exploring CO-tolerant catalysts. To test the electrochemical property of CO-tolerant catalysts, chronoamperometry is widely used under a CO/H_2_ mixture gas atmosphere as an essential method. However, in most cases of catalysts with high CO tolerance, the conventional chronoamperometry has difficulty in showing the apparent performance difference. In this study, we propose a facile and precise test protocol to evaluate the CO tolerance via a combination of short-term chronoamperometry and a hydrogen oxidation reaction (HOR) test. The degree of CO poisoning is systematically controlled by changing the CO adsorption time. The HOR polarization curve is then measured and compared with that measured without CO adsorption. When the electrochemical properties of PtRu alloy catalysts with different atomic ratios of Pt to Ru are investigated, contrary to conventional chronoamperometry, these catalysts exhibit significant differences in their CO tolerance at certain CO adsorption times. The present work will facilitate the development of catalysts with extremely high CO tolerance and provide insights into the improvement of electrochemical methods.

## 1. Introduction

Polymer electrolyte membrane fuel cells (PEMFCs) that use hydrogen (H_2_) as a fuel have attracted increasing research attention as the most promising next-generation power sources because of an eco-friendly reaction mechanism and high power density [1,2,3]. Conventionally, H_2_ is produced by steam reforming of methane gas, and the produced H_2_ gas contains trace amounts of carbon monoxide (CO) of 10–100 ppm [4,5]. However, this small amount of carbon monoxide is sufficient to cause detrimental effects on Pt catalysts widely used in PEMFCs, causing a tremendous suppression in their catalytic performance [6,7]. To alleviate the effect of CO poisoning, various strategies have been adopted, including alloying of Pt with other transition metals, such as Ru, Fe, and Co, formation of core-shell structures, and modification of support materials [8,9,10,11]. Among these strategies, the alloying of Pt with transition metals has been regarded as an efficient way to enhance the CO tolerance of Pt-based catalysts. Especially, PtRu catalysts have exhibited interesting electrochemical properties towards various anodic reactions, such as methanol and CO oxidation reactions, due to bi-functional and ligand effects, resulting in even higher CO tolerance in the hydrogen oxidation reaction (HOR) using reformed H_2_ gas [12,13,14,15,16].

In general, the CO tolerance for the HOR catalysts is evaluated using a CO/H_2_ mixture gas (CO concentrations are 10–100 ppm) as a fuel in a half-cell. In addition, the most commonly used electrochemical method is chronoamperometry, by which the change in currents can be observed as a function of time at a constant potential [17,18]. The chronoamperometry test has the advantage of observing the change in the HOR current at room temperature since only small amounts of catalysts are evaluated on a glassy carbon electrode. However, conventional chronoamperometry has difficultly in distinguishing the superiority of catalysts with relatively high CO tolerance, despite the physical characteristics (surface structure and composition) of the catalysts being different [19,20,21,22,23,24]. For instance, if a novel catalyst with a much higher CO tolerance is designed and surpasses the CO tolerance of the PtRu catalyst, the chronoamperometry technique cannot be a useful method in identifying the novelty of the developed catalysts since the performance difference is not very divergent in the chronoamperogram [24]. Therefore, to supplement the chronoamperometry results, researchers usually conduct additional characterization, such as CO stripping and methanol oxidation reaction (MOR) tests. Otherwise, one must simultaneously increase the CO concentration (200~500 ppm) in the CO/H_2_ mixture gas and the reaction temperature (40~60 °C) for the chronoamperometry test. Alternatively, after fabricating a membrane electrode assembly (MEA) for a unit cell test, its current–voltage (I–V) curve can be measured using the CO/H_2_ mixture gas as fuel. However, in this case, it takes a long time to make the MEA, and a skillful MEA fabrication technique is additionally required. Furthermore, if the concentration of carbon monoxide is too small (e.g., 10–100 ppm), the performance difference remains negligible since much larger amounts of catalysts are used in the MEA compared to the catalyst loading on the glassy carbon for the half-cell test. Therefore, there is a need to develop a unique and simple catalyst evaluation technique that can demonstrate distinguishable CO tolerance and clearly identify the superiority among the catalysts with much higher CO tolerance even at room temperature using a half-cell test with few experimental variables. 

In this work, we propose a facile and reliable test protocol to evaluate the CO poisoning via a combination of short-term chronoamperometry and a simple HOR test. After pre-adsorption of CO on catalysts at a certain time during the short-term chronoamperometry, the HOR polarization curve was simply measured at room temperature and compared with that measured without CO adsorption to corroborate the CO tolerance. To confirm the applicable feasibility of the protocol, the electrochemical properties of PtRu alloy (Pt_1_Ru_1_ and Pt_1_Ru_3_) catalysts with different atomic ratios of Pt to Ru were mainly investigated. Through the modified tests, the significant difference in their CO tolerance was revealed, while there was no performance difference between them in the conventional chronoamperometry tests. Therefore, we believe that this protocol can clearly identify the CO tolerance difference by controlling the adsorption time using a high concentration of CO and effectively monitoring the HOR activity of the catalysts. 

## 2. Materials and Methods 

### 2.1. Materials

All chemical reagents used were of analytical grade and used without further purification. Carbon blacks (Vulcan XC72, Cabot) were purchased from Cabot Inc., Alpharetta, GA, USA. Commercialized Pt_1_Ru_1_/C and Pt/C catalysts (Johnson Matthey) were used as controls. Also, 1-Octadecene (90%), platinum acetylacetonate (Pt(acac)_2_, 97%), ruthenium acetylacetonate (Ru(acac)_2_, 97%), oleylamine (70%), Nafion ionomer (5 wt %), and 2-propanol (99.5%) were procured from Sigma-Aldrich Inc., USA. *n*-Hexane (95%) and ethanol (95%) were acquired from Samchun Pure Chemical, Daejeon, Korea.

### 2.2. Synthesis of Pt_1_Ru_3_/C catalyst

Carbon black (0.1 g) and 3.5 mL of oleylamine were well dispersed through ultrasonication in 147 mL of 1-octadecene for 20 min. Meanwhile, 0.034 g of Pt(acac)_2_, 0.035 g of Ru(acac)_2_, 3.5 mL of oleylamine, and 13 mL of 1-octadecene were taken in a separate vial and allowed to disperse through ultrasonication for 20 min. Afterwards, the two solutions were mixed well and further sonicated for 5 min and subjected to heating at 120 °C for 1 h under an Ar atmosphere to remove H_2_O impurities. After 1 h, the solution temperature was gradually raised to 300 °C and maintained for 2 h. After the thermal decomposition reaction of Pt and Ru precursors, the solution was cooled down to 80 °C. The catalyst-containing solution was filtered and then washed with a copious amount of *n*-hexane and ethanol solution. After the obtained product was dried in a vacuum oven at 60 °C, it was annealed at 600 °C for 2 h in 5% H_2_-mixed N_2_ gas and formulated as a Pt_1_Ru_3_/C catalyst. 

### 2.3. Characterizations

The microstructure and morphologies of the catalysts, Pt_1_Ru_1_/C and Pt_1_Ru_3_/C, were observed using a high-resolution transmission electron microscopy (HRTEM, Tecnai G^2^ F30 S-Twin, FEI Thermo Fisher Scientific, Eindhoven, Netherlands). The atomic composition of the Pt_1_Ru_3_/C catalyst was confirmed by energy-dispersive X-ray spectroscopy (EDX, Tecnai G^2^ F30 S-Twin, FEI Thermo Fisher Scientific, Eindhoven, Netherlands) analysis using the TEM as shown in Figure S1. All electrochemical measurements were tested in a standard three-electrode system using a rotating disk electrode (RDE, Metrohm, Switzerland) with a glassy carbon (GC, Metrohm, Switzerland), Pt wire, and Ag/AgCl electrode as the working, counter, and reference electrodes, respectively. All potential values were represented versus the reversible hydrogen electrode (RHE). The RHE calibration was performed in an electrolyte solution by measuring the current using a Pt disk electrode between the potential ranges of hydrogen oxidation and evolution reactions. The catalyst ink was prepared by dispersing a mixture containing 5 mg of the prepared catalyst, Nafion ionomer (68.7 µL), and 2-propanol (500 µL) through ultrasonication for few minutes. A drop of catalyst ink (total metal loading = 44.86 μg cm^−2^) was applied to a glassy carbon electrode (0.196 cm^2^ geometric surface area) and then dried at room temperature. Cyclic voltammograms (CVs) were measured by cycling the potential between 0.05 and 1.05 V_RHE_ at a scan rate of 20 mV s^−1^ in an Ar-saturated 0.1 M HClO_4_ electrolyte solution. For HOR tests, the potential was applied from −0.05 to 0.1 V_RHE_ at a scan rate of 1 mV s^−1^ under a constant rotation speed of 1600 rpm in H_2_-saturated 0.1 M HClO_4_ solution. For CO stripping measurements, the working electrode potential was held at 0.05 V_RHE_ for 15 min while bubbling pure CO gas into 0.1 M HClO_4_. After purging the electrolyte with Ar gas for 20 min, the residual CO molecules in the electrolyte were completely removed, and then, in Ar-saturated electrolyte, a CV was obtained at a scan rate of 20 mV s^−1^ within the potential range of 0.05 and 1.05 V_RHE_. The electrochemically active surface area (ECSA) was calculated by integrating the current in the CO oxidation peak region assuming a monolayer adsorption of CO molecules. For all chronoamperometry tests, the potential was maintained at 0.05 V_RHE_ for 7200 s at 1600 rpm in H_2_- and 100 ppm CO-mixed H_2_-saturated 0.1 M HClO_4_. For the proposed new CO tolerance test, conventional chronoamperometry was modified by systematically controlling the gas atmosphere. First, the conventional chronoamperometry was performed for ~60 s in the same condition (at 0.05 V_RHE_ with a rotation speed of 1600 rpm) using H_2_-saturated 0.1 M HClO_4_. After ~60 s, the gas atmosphere was immediately changed from H_2_ to CO while maintaining the applied potential (0.05 V_RHE_). In this step, pure CO gas was bubbled into 0.1 M HClO_4_ for a period of time (∆t_CO_ = 0, 5, 15, or 30 s) for partial adsorption of CO molecules on the catalyst surface, followed by purging H_2_ gas for 20 min. Finally, the HOR polarization curve was measured in H_2_-saturated 0.1 M HClO_4_ at a scan rate of 1 mV s^-1^ within the potential range of −0.05 to 0.1 V_RHE_ under a constant rotation speed of 1600 rpm.

## 3. Results and Discussion

The main purpose of this work is to provide clear insights into the facile and precise evaluation of CO tolerance for catalysts with different electrochemical properties. Therefore, to emphasize the applicable feasibility of the test protocol for CO tolerance measurement, we compared the electrochemical properties of two representative carbon-supported PtRu (Pt_1_Ru_1_/C and Pt_1_Ru_3_/C) alloy catalysts composed of different atomic ratios of Pt to Ru. First of all, the Pt_1_Ru_3_/C catalyst was prepared by a solution-based thermal decomposition reaction (the detailed synthesis procedure was described in the Section 2), and its CO tolerance was then compared with that of commercialized Pt_1_Ru_1_/C and Pt/C catalysts as controls. 

It is well known that alloying Ru with Pt enhances the CO tolerance of the active Pt surface for the HOR by decreasing the CO binding energy [13]. For the Pt_1_Ru_1_/C catalyst, it is widely reported that the equivalent content of Pt and Ru (Pt/Ru = 1:1) is an ideal atomic ratio to have appropriately low CO binding energy and the number of active Pt sites can be ensured [25]. On the other hand, in the case of the Pt_1_Ru_3_/C catalyst, slightly lower CO tolerance is expected, since increasing the ratio of Pt to Ru above 1 causes an increase in CO binding energy of PtRu alloys [25,26].

**Figure 1:**
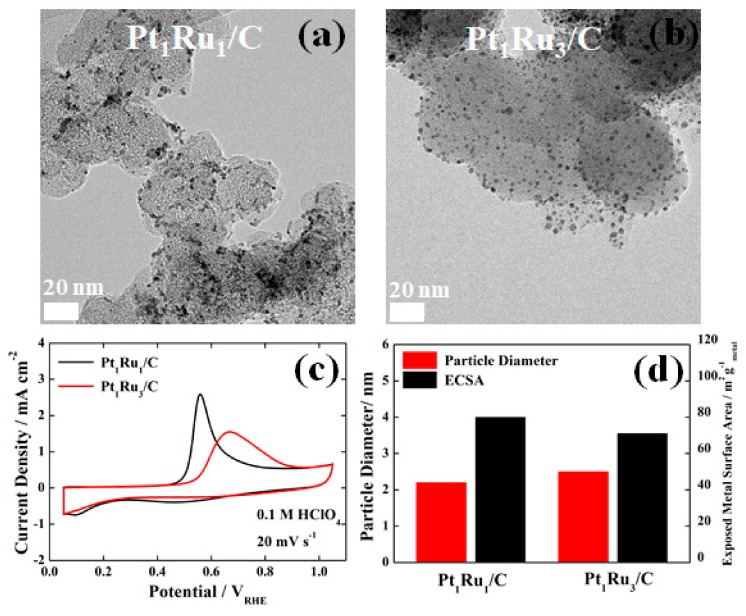
Transmission electron microscopy (TEM) images of (**a**) Pt_1_Ru_1_/C and (**b**) Pt_1_Ru_3_/C, (**c**) CO-stripping curves and (**d**) particle diameters and electrochemically active surface areas (ECSAs) of Pt_1_Ru_1_/C and Pt_1_Ru_3_/C.

As shown in Figure 1a,b, TEM images of Pt_1_Ru_1_/C and Pt_1_Ru_3_/C clearly show the uniform distribution of PtRu alloy nanoparticles with a particle size of 2 to 3 nm on carbon supports. To further understand the difference in the surface properties of Pt_1_Ru_1_/C and Pt_1_Ru_3_/C catalysts, CO stripping tests were performed for each sample, as shown in Figure 1c. The ECSA of a sample is obtained by integrating CO oxidation currents, and the information about its electrochemical property can be qualitatively estimated from the onset and peak potentials for the CO oxidation. As expected, the onset and peak potentials (0.45 and 0.57 V_RHE_, respectively) for the CO oxidation of Pt_1_Ru_1_/C were much lower than those (0.57 and 0.66 V_RHE_, respectively) of Pt_1_Ru_3_/C, indicating higher CO oxidation activity than Pt_1_Ru_3_/C [26]. However, it was clearly confirmed that the two PtRu/C catalysts still exhibit much higher CO oxidation activities due to the presence of surface Ru atoms compared to a bare Pt/C catalyst [27]. This result corresponded to that of previous reports showing that the change in the CO binding energy depends on the Ru content in PtRu alloys [13].

However, the CO stripping (or CO oxidation) test results, which were obtained by a potential scan between 0.05 and 1.05 V_RHE_, cannot directly represent the CO tolerance characteristic of the samples in the HOR since the anode overpotential is practically limited within 0.2 V_RHE_ in PEMFCs [28,29]. Meanwhile, as shown in Figure 1d, Pt_1_Ru_1_/C and Pt_1_Ru_3_/C had similar particle diameters (estimated from the TEM images in Figure 1a,b) and ECSA values (calculated from the CO stripping curves shown in Figure 1c). From the above results, it might be concluded that Pt_1_Ru_1_/C and Pt_1_Ru_3_/C catalysts have a similar particle size and surface area while they have different CO binding energies due to different Ru content.

**Figure 2:**
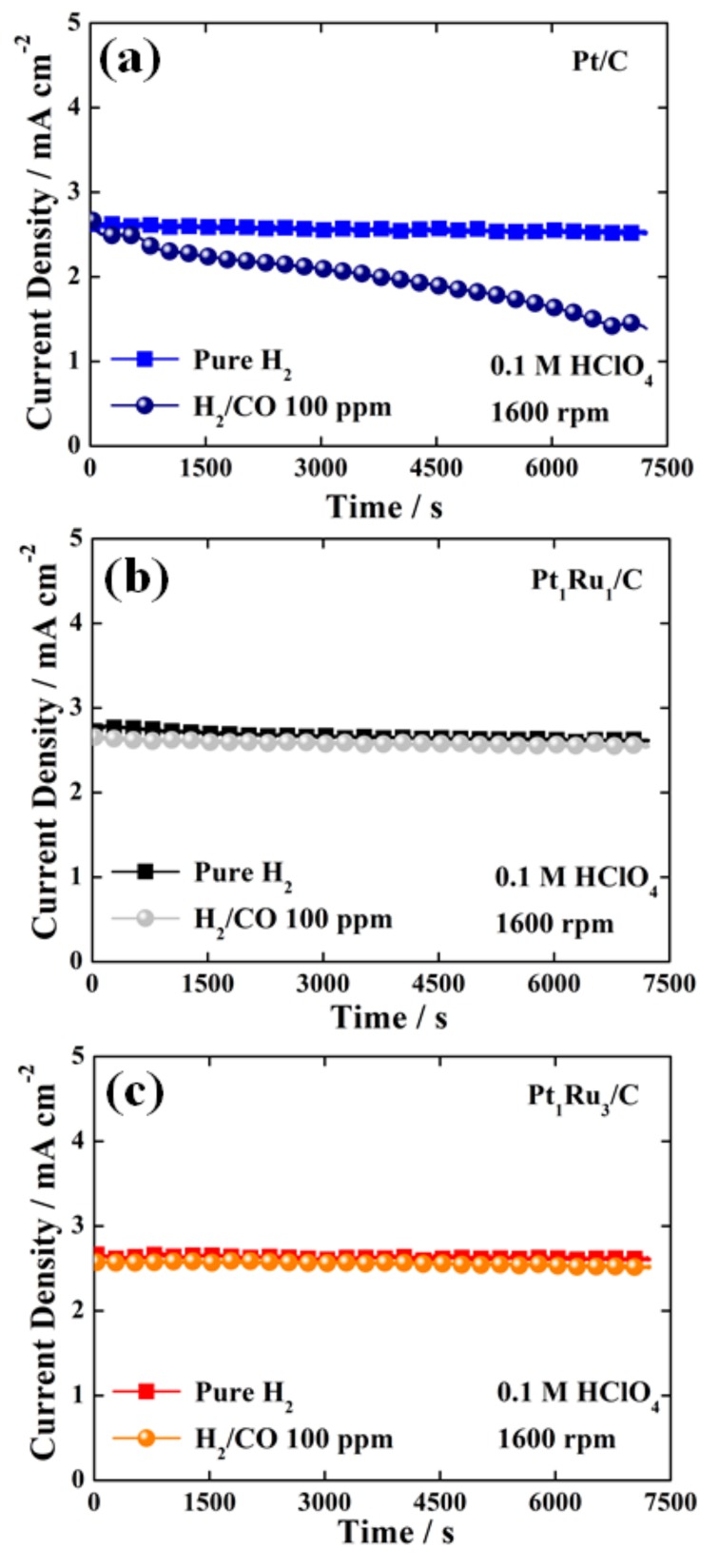
Chronoamperograms of (**a**) Pt/C, (**b**) Pt_1_Ru_1_/C, and (**c**) Pt_1_Ru_3_/C measured in H_2_- and 100 ppm CO-mixed H_2_-saturated 0.1 M HClO_4_ at a constant potential of 0.05 V_RHE_ for 7200 s.

To investigate the CO tolerance of the catalysts, a chronoamperometry test, one of the most commonly used evaluation techniques, was conducted. Definitely, a Pt/C catalyst without Ru indicated much lower CO tolerance in the chronoamperometry tests, changing the gas atmosphere from pure H_2_ to 100 ppm CO-mixed H_2_ gases, as shown in Figure 2a. In sharp contrast, it was difficult to determine which of the Pt_1_Ru_1_/C and Pt_1_Ru_3_/C catalysts had better CO tolerance in the same test condition. As shown in Figure 2b and c, in pure H_2_ and 100 ppm CO-mixed H_2_ gas atmospheres, both Pt_1_Ru_1_/C and Pt_1_Ru_3_/C catalysts exhibited similarly high CO tolerance characteristics although they showed different CO oxidation activity in the CO stripping tests, as shown in Figure 1c. Hence, it is reasonable to assume that the above conventional chronoamperometry is not sensitive enough to judge the superiority between PtRu-based catalysts having a much lower CO binding energy and sufficiently high CO tolerance. 

Alternatively, the test might be carried out using CO/H_2_ mixture gases containing more CO than 100 ppm to monitor the performance differences [30]. However, if CO/H_2_ mixture gases with a much higher CO concentration are used for the test at room temperature, it might be difficult to obtain normal HOR currents even for PtRu catalysts since the amount of CO adsorption on the Pt surface is considerably increased. Therefore, simply increasing the CO concentration in CO/H_2_ mixture gas is not helpful to clearly confirm the difference in the CO tolerance of catalysts at room temperature in half-cell tests using a very small catalyst loading on a glassy carbon electrode. To minimize the effect of temperature on the evaluation quality, in most cases, CO tolerance tests using CO/H_2_ mixture gases with a high CO concentration have been performed in unit cells operating at high temperature (60–85 °C) [31]. However, in this case, one has to fabricate an MEA and conduct the activation process of the MEA for a long time before the CO tolerance test. Therefore, it is crucial to develop a facile and reliable evaluation protocol that provides accurate and significant differences in CO tolerance testing even at room temperature in a half-cell.

**Figure 3:**
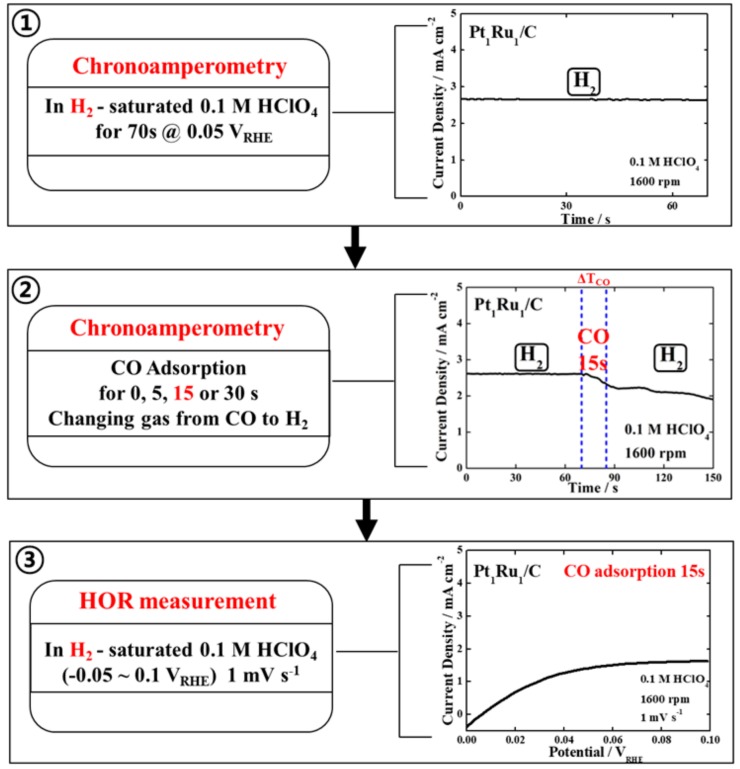
Proposed test protocol to evaluate the CO tolerance and the test results of the Pt_1_Ru_1_/C catalyst as an example in each step. Step (**1**) chronoamperometry of catalyst in H_2_-saturated 0.1 M HClO_4_ at a constant potential of 0.05 V_RHE_ for ~60 s, Step (**2**) 15 s CO adsorption on catalyst followed by H_2_ purging for 20 min at a constant potential of 0.05 V_RHE_, and Step (**3**) hydrogen oxidation reaction (HOR) polarization curve of catalyst in H_2_-saturated 0.1 M HClO_4_.

As shown in Figure 3, we designed a facile test protocol that combines short-term chronoamperometry and a simple HOR test at room temperature as an alternative approach. The proposed test protocol entails the following order of analysis. Step (1)—after purging the electrolyte with H_2_ gas for 20 min, measure chronoamperometry at a constant potential of 0.05 V_RHE_ for ~60 s in H_2_-saturated electrolyte to confirm the HOR current of a sample under normal operating conditions, as shown in Figure S2a. Step (2)—change the bubbling gas from H_2_ to 100% CO gas to poison the catalyst surface with CO molecules for a desired time, e.g., 15 s, while simultaneously monitoring the changes in the chronoamperogram, as shown in Figure S2b–d. After the CO poisoning time, change the bubbling gas back to pure H_2_ gas. Step (3)—after purging H_2_ gas for 20 min in the electrolyte, measure the HOR polarization curve in the potential range of -0.05 to 0.1 V_RHE_. The HOR polarization curve measured in the last step can be compared with that measured in a pure H_2_ atmosphere without CO adsorption (0 s) to corroborate the CO tolerance.

**Figure 4:**
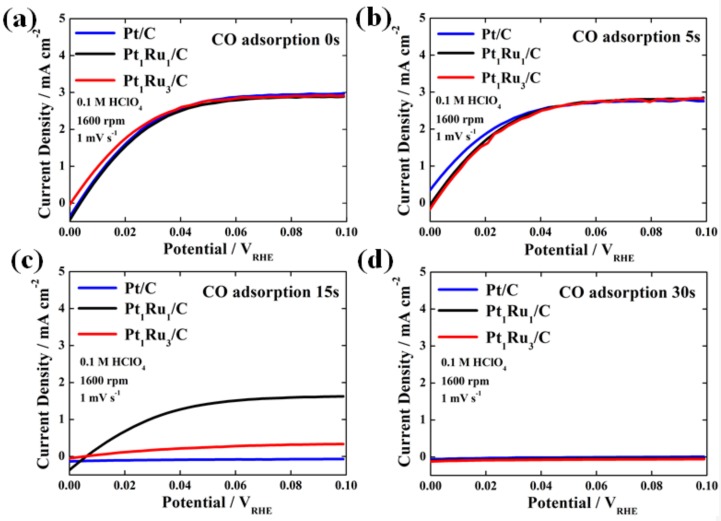
The HOR polarization curves of commercial Pt/C, Pt_1_Ru_1_/C, and Pt_1_Ru_3_/C catalysts measured in H_2_-saturated 0.1 M HClO_4_ after the CO adsorption for (**a**) 0 s (without CO adsorption), (**b**) 5 s, (**c**) 15 s, and (**d**) 30 s.

The HOR polarization curves for the prepared catalysts depending on the CO adsorption time are presented in Figure 4a–d. In Figure 4a, it is noted that the HOR curves of Pt/C, Pt_1_Ru_1_/C, and Pt_1_Ru_3_/C catalysts without CO adsorption show no noticeable difference. Similarly, when the CO adsorption time is 5 s in Step (2) shown in Figure 3, their HOR performances hardly changed because the CO adsorption time might have been too short, as shown in Figure 4b. It can be regarded as insufficient CO poisoning time in decreasing the performance of the catalysts. Interestingly, as shown in Figure 4c, we observed a clear-cut difference in performance between the two PtRu catalysts after CO was adsorbed onto the catalysts for 15 s in Step (2), although the HOR current of Pt/C without CO tolerance was zero. Notably, after 15 s of CO adsorption, the Pt_1_Ru_1_/C catalyst with an atomic ratio of 1:1 exhibited a ~50% reduction in the HOR current density, while Pt_1_Ru_3_/C showed near-zero tolerance toward CO, and the current density was drastically reduced, which is mainly attributed to severe catalyst poisoning. This result supports lower CO binding energy (higher CO oxidation reaction activity) of Pt_1_Ru_1_/C, confirmed by the CO stripping analysis, as shown in Figure 1c, and proves that Pt_1_Ru_1_ alloy has much higher CO tolerance compared to Pt_1_Ru_3_ [25,26]. Furthermore, when we increased the CO adsorption time to 30 s in Step (2), Pt_1_Ru_1_/C, as well as Pt_1_Ru_3_/C and Pt/C, indicated no HOR current since most active sites in both samples were completely poisoned by CO molecules, as shown in Figure 4d. 

Consequently, it is worth noting that, in our evaluation protocol, 15 s was the optimum time for the CO adsorption to clearly confirm the difference in electrochemical properties of catalysts with low CO binding energy and sufficiently high CO tolerance. To further ascertain that 15 s CO adsorption is the optimum time clearly demonstrating the CO tolerance of PtRu catalysts, CO stripping analyses of all catalysts with different CO adsorption times were conducted again after the HOR tests and the corresponding CO coverage was calculated from the results, as shown in Figure S3. To investigate the change in CO coverage, the CO oxidation peak area obtained after 900 s of CO adsorption for each sample was served as a standard area (100%) with the catalyst surface completely covered by CO. From the CO stripping analyses, it was revealed that the CO coverage on the catalyst surface was controlled depending on the CO binding energy. As expected, in the case of CO adsorption for 15 s as the optimum value, the difference in CO coverage among the samples was apparent. 

## 4. Conclusions

A facile and accurate test protocol, which combines short-term chronoamperometry and a simple HOR test, was proposed to evaluate the CO tolerance of catalysts with low CO binding energy and sufficiently high CO tolerance at room temperature. In this protocol, the degree of the catalyst poisoning was systematically controlled by changing the CO adsorption time with 100% CO gas. After the CO adsorption, the HOR polarization curve was obtained and compared with that measured in a pure H_2_ atmosphere without CO. To prove the application feasibility of the proposed evaluation protocol, the electrochemical properties of two representative PtRu (Pt_1_Ru_1_/C and Pt_1_Ru_3_/C) alloy catalysts with different atomic ratios of Pt to Ru were intensively studied. The conventional chronoamperometry showed no drastic difference in CO tolerance in both samples since they have sufficiently low CO binding energy and high CO tolerance as evidenced from the CO stripping curves and chronoamperograms, respectively. In sharp contrast, using the proposed test protocol, the CO tolerance of the catalysts were sensitively changed depending on the CO adsorption time. Especially, when the CO adsorption time was 15 s (the optimum condition), the HOR polarization curves of Pt_1_Ru_1_/C and Pt_1_Ru_3_/C showed a noticeable difference in CO tolerance even at room temperature. Accordingly, we believe that the present work will support the development of a wide variety of Pt-based alloy catalysts showing high CO tolerance and may further provide insights into the improvement of conventional electrochemical methods.

## Supplementary Materials

The following are available online at https://www.mdpi.com/2079-4991/9/10/1425/s1, Figure S1: TEM image, elemental maps of Pt and Ru, and the corresponding EDX spectrum of (a) Pt1Ru1 and (b) Pt1Ru3 nanoparticles; Figure S2: Chronoamperograms of Pt/C, Pt1Ru1/C, and Pt1Ru3/C measured switching a bubbling gas (H2 and 100% CO gases) in 0.1 M HClO4 at a constant potential of 0.05 VRHE for 150 s at a rotation speed of 1600 rpm. For the CO adsorption on the catalyst surface, 100% CO was supplied into the electrolyte for (a) 0 s, (b) 5 s, (c) 15 s, and (d) 30 s; Figure S3: CO stripping curves of (a) Pt/C, (c) Pt1Ru1/C, and (e) Pt1Ru3/C measured after the CO adsorption for 5, 15, 30, and 900 s. Change in the CO coverage (%) on the catalyst surfaces for (b) Pt/C, (d) Pt_1_Ru_1_/C, and (f) Pt_1_Ru_3_/C estimated from the corresponding CO stripping curves.
