# Supplementary material for: Electrochemical Analysis for Demonstrating CO Tolerance of Catalysts in Polymer Electrolyte Membrane Fuel Cells
Source: Nanomaterials (Basel). 2019 Oct 8;9(10):1425. doi: 10.3390/nano9101425 (PMC6835550; doi:10.3390/nano9101425)
Supplement: Supplementary file 1 [file nanomaterials-09-01425-s001.pdf]

Supporting Information for

Electrochemical Analysis for Demonstrating CO Tolerance of Catalysts in Polymer Electrolyte Membrane Fuel Cells

Jiho Min [1†], A. Anto Jeffery [1†], Youngjin Kim [1] and Namgee Jung [1,*]

[1] Graduate School of Energy Science and Technology (GEST), Chungnam National University, 99 Daehak-ro, Yuseong-gu, Daejeon, 34134, Republic of Korea; mjh9780@naver.com (J.M.); jeffeeanto@gmail.com (A.A.J.); yoongjin123@naver.com (Y.J.)

* Correspondence: njung@cnu.ac.kr (N.J.)

† These authors contributed equally to this work.

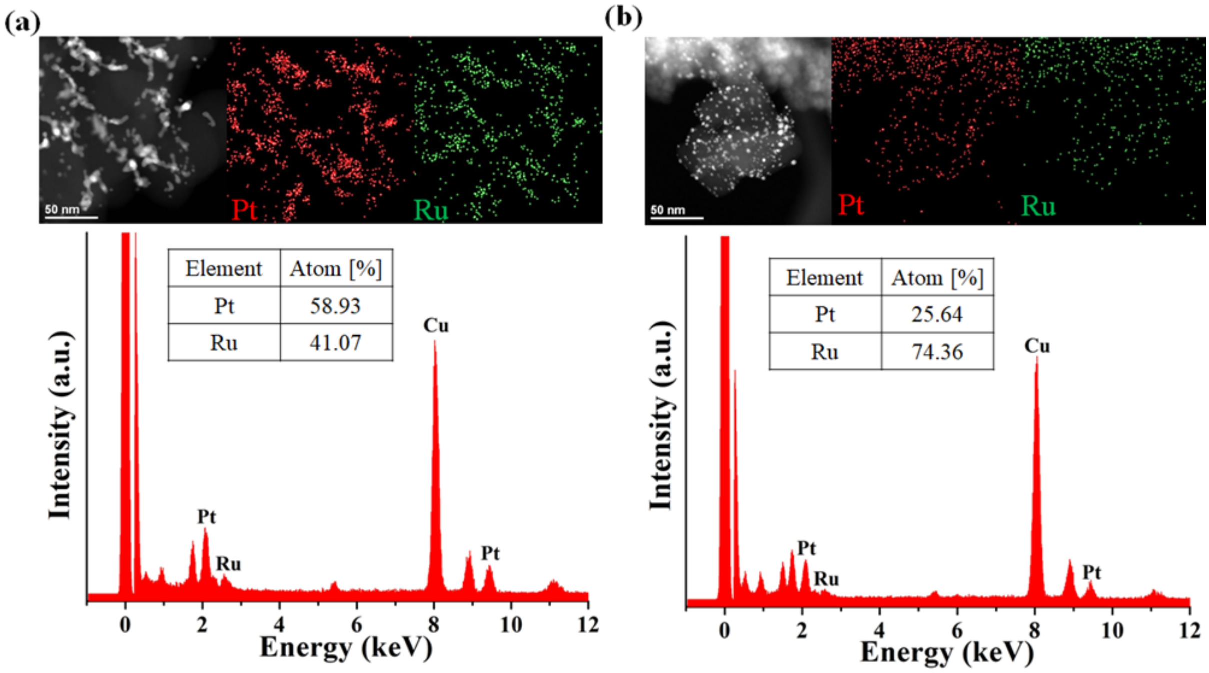

Figure S1. TEM image, elemental maps of Pt and Ru, and the corresponding EDX spectrum of (a) Pt_1Ru_1 and (b) Pt_1Ru_3 nanoparticles.

Elemental analysis of Pt and Ru within PtRu nanoparticles were examined by energy-dispersive X-ray spectrocopy (EDX). For both the catalysts, Pt_1Ru_1 and Pt_1Ru_3, elemental distributions of Pt and Ru reveal uniform distribution of Pt and Ru within PtRu nanoparticles. As shown in the EDX spectra (Figure S1a and b), the characteristic peaks of Pt and Ru further confirms uniform distribution of all elements and no other contaminants were seen. The difference between two catalysts (Pt_1Ru_1 and Pt_1Ru_3) from the elemental mapping and EDX spectra cannot be accurately determined due to the fact that the element signals from different areas of scanning show moderate variations.

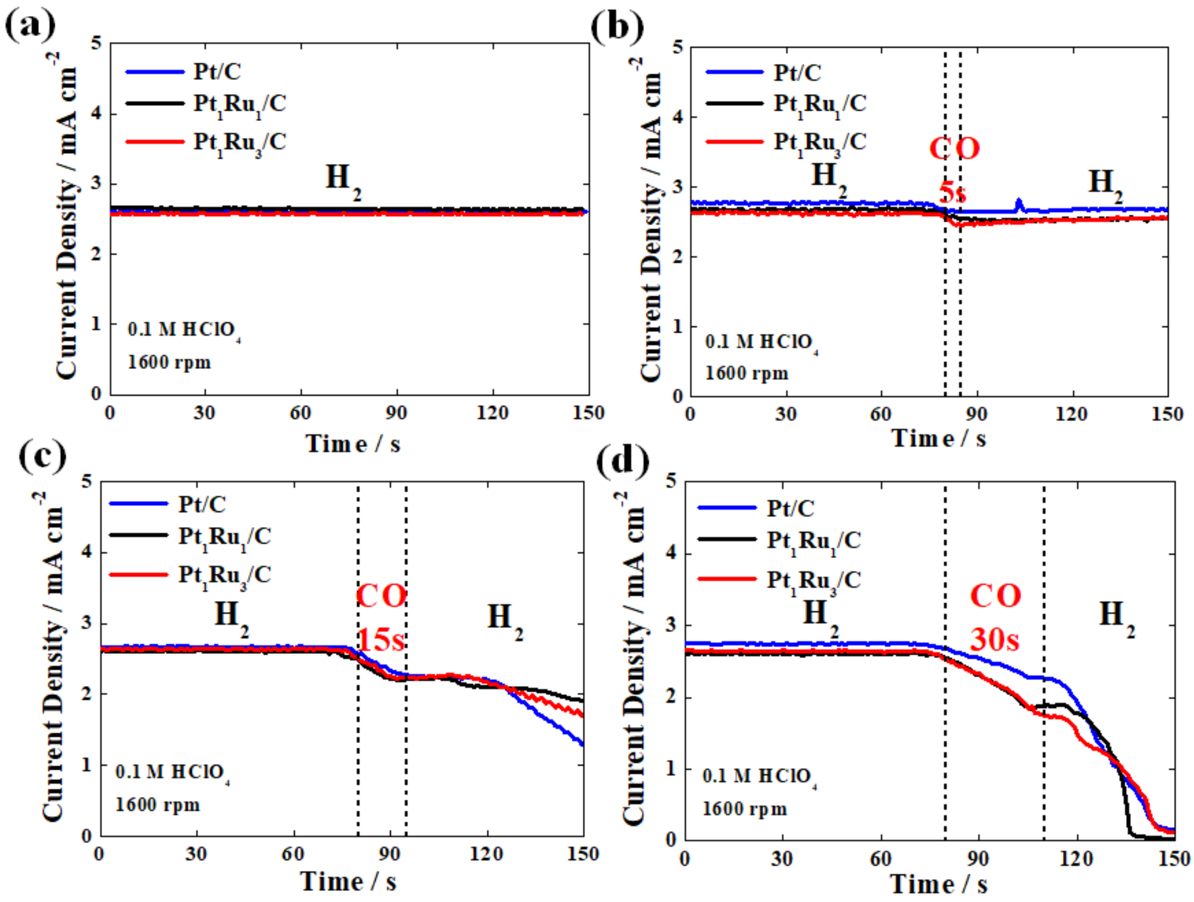

Figure S2. Chronoamperograms of Pt/C, Pt_1Ru_1/C, and Pt_1Ru_3/C measured switching a bubbling gas (H_2 and 100 % CO gases) in 0.1 M $HClO_4$ at a constant potential of 0.05 V_{RHE} for 150 s at a rotation speed of 1600 rpm. For the CO adsorption on the catalyst surface, 100 % CO was supplied into the electrolyte for (a) 0 s, (b) 5 s, (c) 15 s, and (d) 30 s.

Figure S2 displays chronoamperograms of Pt_1Ru_1/C and Pt_1Ru_3/C compared with Pt/C for the proposed test protocol in step (2) of Figure 4. As shown in Figure S2a, for 0 s CO adsorption, all the catalysts showed no change in the current density. After 5 s of CO adsorption, the Pt_1Ru_1/C and Pt_1Ru_3/C catalysts exhibited similar chronoamperogram with only a slight decrease in the current density, that indicates very little poisoning by CO molecules (Figure S2b). When the CO gas adsorption was increased to 15 s, which is regarded as the optimum condition of our proposed test protocol, it is reasonable to make a direct comparison among the catalysts as shown in Figure S2c. As expected, Pt_1Ru_1/C showed higher current density compared to Pt_1Ru_3/C and Pt/C after the CO adsorption. However, the current density of Pt_1Ru_3/C catalyst was slightly less than that of Pt_1Ru_1/C and much higher than that of Pt/C. Furthermore, for 30 s of CO adsorption, all the catalysts showed sharp decrease in the current density, which suggests severe catalyst poisoning by CO molecules.

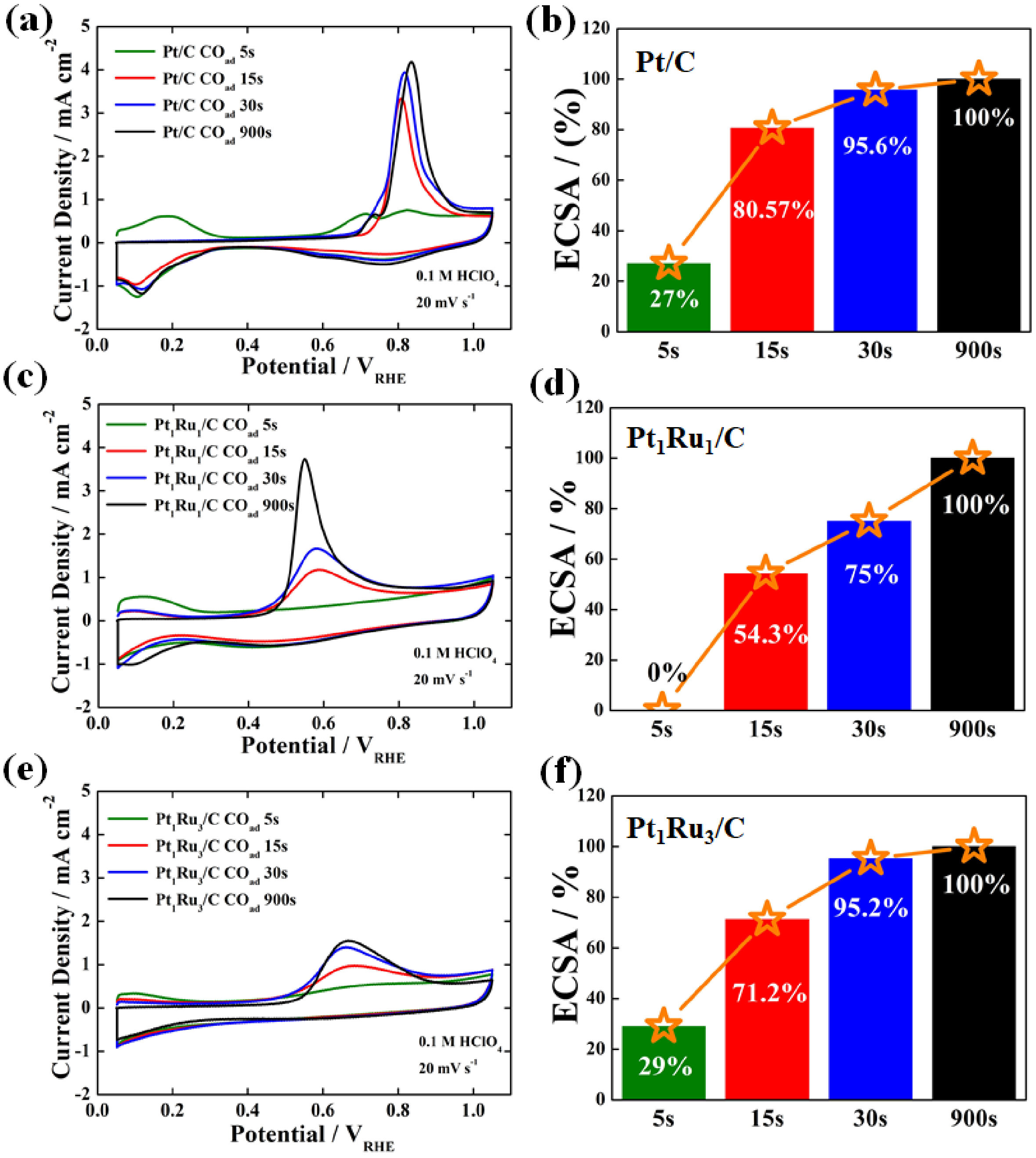

Figure S3. CO stripping curves of (a) Pt/C, (c) Pt_1Ru_1/C, and (e) Pt_1Ru_3/C measured after the CO adsorption for 5, 15, 30, and 900 s. Change in the CO coverage (%) on the catalyst surfaces for (b) Pt/C, (d) Pt_1Ru_1/C, and (f) Pt_1Ru_3/C estimated from the corresponding CO stripping curves.
